# Idiopathic Peripheral Neuropathy Responsive to Sympathetic Nerve Blockade and Oral Clonidine

**DOI:** 10.1155/2012/407539

**Published:** 2012-08-26

**Authors:** Jenna L. Walters, Daniel F. Lonergan, Robert D. Todd, Tracy P. Jackson

**Affiliations:** Department of Anesthesia, Vanderbilt University Medical Center, Nashville, TN 37212, USA

## Abstract

A 52-year-old female presented with idiopathic stocking-glove neuropathy. She underwent a series of right and left stellate ganglion blocks with ropivacaine and clonidine, followed by lumbar sympathetic blocks. This resulted in complete symptom relief for two weeks. These procedures were repeated after a two-month interval; at that time she was still experiencing partial relief from the first series. She again remained completely pain free for several weeks following the injections. As the pain partially returned, daily oral clonidine was initiated and resulted in almost complete cessation of her symptoms, which persisted at a three-month follow-up examination.

## 1. Introduction

Peripheral neuropathy can be a debilitating neurologic disease that presents a challenge in chronic pain management. The prevalence of peripheral neuropathy has been reported in the general population to range from 2.4 to 8.0 percent with a higher prevalence in the elderly [1]. Patients usually present with complaints of burning pain in the distal upper and lower extremities [1]. They may also experience numbness or tingling, weakness, hyporeflexia, muscle atrophy, or hypersensitivity in the distribution of their pain [1, 2]. The differential diagnosis for this disease is broad and includes inherited disorders and a host of chronic illnesses such as diabetes and leprosy [1, 2]. Other causes of peripheral neuropathy include traumatic injury, vitamin deficiency, infection, toxins, and medication side effects [1, 2]. The medical testing required to differentiate between these causes can be time consuming and may still not reveal a diagnostic cause in up to 25 percent of patients [1].

## 2. Case Description

The patient was a 52-year-old female with a history of bipolar disorder and smoking who presented with idiopathic stocking-glove neuropathy. Prior to the onset of her symptoms she had undergone a C5-C6 anterior fusion for cervical radiculopathy. A complete rheumatologic workup to determine the cause of her neuropathy was negative. An electromyogram showed no evidence of radiculopathy or neuropathy. A cervical MRI revealed chronic flattening of the spinal cord, but the patient had no clinical evidence of myelopathy such as sensory or motor deficits on physical exam. Previous treatment strategies included unsuccessful carpel tunnel and trigger thumb release. On initial presentation her medications included methadone 10 mg twice daily and oxycodone 60 mg four times daily as needed; however, she continued to rate her pain as a 9 out of 10 in severity. 

The patient was originally treated with a right stellate ganglion block containing ropivacaine and clonidine. This was performed under fluoroscopic guidance by injecting local anesthetic at the junction of the C6 uncinate process and vertebral body [3], creating a sympathectomy to the head, neck, and upper extremities. This intervention resulted in complete symptom relief in her right upper extremity for two weeks. The patient then underwent left stellate ganglion block with similar results in her left-upper extremity. 

Given the successful treatment of her upper extremity peripheral neuropathy with sympathetic blockade, a trial of lumbar sympathetic blockade was initiated in her lower extremities. Lumbar sympathetic blockade was performed by injection at the anterolateral surface of L2-L3 vertebral bodies [4]. Each side of the sympathetic blockade was separated by a time frame of approximately two weeks. Both lumbar sympathetic blocks produced symptom relief for approximately two weeks similar to the upper extremity blockade. These procedures were repeated after a two-month interval. At that time, she was still experiencing partial relief from the first series. She again remained completely pain free for several weeks following the injections.

As the pain partially returned, pregabalin was added to her medication regimen. However, she experienced sedation and dizziness which required discontinuation. Twice daily oral clonidine 0.1 mg was then initiated and resulted in almost complete cessation of her symptoms of peripheral neuropathy. 

## 3. Discussion

Sympathetic nerve blockade of the upper and lower extremities is the treatment of choice for severe, sympathetically mediated pain that cannot be managed appropriately with oral regimens [5] and serves as a diagnostic and therapeutic intervention for sympathetically mediated pain [4–6]. A specific type of sympathetic block, stellate ganglion blockade, is used for many chronic pain syndromes that involve the head and upper extremities [5, 6]. It has been used to treat complex regional pain syndrome (CRPS) I and II, vascular insufficiency, lymphedema, postherpetic neuralgia, phantom limb pain, acute hearing loss, hyperhidrosis, vascular-mediated headache and neuropathic pain [5, 6]. The indications for lumbar sympathetic blockade are similar but involve pain distributed in the lower extremities. They include CRPS of the lower extremities, phantom limb pain, postherpetic neuralgia, and vascular insufficiency including peripheral arteriosclerotic disease [4, 5].

The patient had a more prolonged response to sympathetic blockade than expected given the rapid metabolism of local anesthetics and clonidine. This suggests that a brief interruption of the sympathetic chain may have a prolonged effect on sympathetic neural firing. In addition to sympathetic blockade, a reduction in sympathetic tone by adding clonidine to her medical regimen seemed to have a durable therapeutic effect on her sympathetically mediated pain. 

Clonidine is an alpha-2 agonist originally used in the treatment of hypertension which has recently gained popularity in the treatment of perioperative and chronic pain. Clonidine's role as an analgesic is based on experimental studies showing that it augments hyperpolarization based on the frequency of neuronal firing [7]. The addition of clonidine to prolong peripheral nerve blockade has been demonstrated in several clinical trials [7]. In recent studies, doses of systemic clonidine in 4 *μ*g/kg have been shown to decrease opioid requirement without significant cardiovascular effects [7]. The role of clonidine in chronic pain management has been less studied but may play a role as an adjuvant to more traditional medical regimens.

The treatment of idiopathic peripheral neuropathy can present a challenge to the pain physician. This case highlights the role of sympathetic blockade and oral pain regimens focused on treating sympathetically mediated pain. If the physician suspects that these debilitating symptoms have a sympathetic component based on the inciting event or patient history, treatment with sympathetic blockade and clonidine may be of benefit when more traditional medical management alone is not sufficient. 
